# Chromoendoscopy to Detect Early Synchronous Second Primary Esophageal Carcinoma in Patients with Squamous Cell Carcinomas of the Head and Neck?

**DOI:** 10.1155/2013/236264

**Published:** 2013-03-20

**Authors:** Pavel Komínek, Petr Vítek, Ondřej Urban, Karol Zeleník, Magdaléna Halamka, David Feltl, Jakub Cvek, Petr Matoušek

**Affiliations:** ^1^Department of Otorhinolaryngology, University Hospital Ostrava, 708 52 Ostrava, Czech Republic; ^2^Faculty of Medicine, University of Ostrava, 703 00 Ostrava, Czech Republic; ^3^Department of Internal Medicine, City Hospital Frýdek-Místek, 738 01 Frýdek-Místek, Czech Republic; ^4^Department of Gastroenterology, Vítkovice Hospital Ostrava, 703 00 Ostrava, Czech Republic; ^5^Department of Oncology, University Hospital Ostrava, 708 52 Ostrava, Czech Republic

## Abstract

*Objective*. To evaluate the use of flexible esophagoscopy and chromoendoscopy with Lugol's solution in the detection of early esophageal carcinomas (second primary carcinomas) in patients with squamous cell carcinoma of the head and neck (HNSCC). *Methods*. All patients with newly diagnosed HNSCC underwent office-based Lugol's chromoendoscopy. After flexible esophagoscopy with white light, 3.0% Lugol's iodine solution was sprayed over the entire esophageal mucosa. Areas with less-intense staining (LVLs) were evaluated and biopsies taken. *Results*. 132 patients with HNSCC were enrolled in this study. The most frequent primary tumors were oropharyngeal (49/132), tumors of the oral cavity (36/132), and larynx (35/132). The majority of subjects (107/132 patients, 81.1%) had advanced HNSCC carcinomas (stages III and IV). Multiple LVLs were discovered in 24 subjects (18.2%) and no LVLs in 108 (81.8%) subjects. Fifty-five LVL biopsy specimens were obtained and assessed. Squamous cell carcinomas were detected in two patients, peptic esophagitis in 11 patients, gastric heterotopic mucosa in two patients, hyperplasia in two patients, and low- and high-grade dysplasia in three patients. *Conclusion*. Although only two patients with synchronous primary carcinomas were found among the patients, esophagoscopy should be recommended after detection of HNSCC to exclude secondary esophageal carcinoma or dysplasia.

## 1. Introduction

Patients with squamous cell carcinomas of the head and neck (HNSCC) region show a predisposition to developing second primary squamous cell carcinomas in the aerodigestive tract [1–3]. While the risk of the existence of a second primary tumor in another area of the head or neck varies from 16% to 36%, the incidence of esophageal squamous cell carcinoma (SESCC) in patients with HNSCC varies from 1% to 17% [3, 4].

Due to the aggressive nature of esophageal cancer and advanced disease at the time of diagnosis, the prognosis of esophageal cancer is generally poor [3, 5, 6]. Therefore, the identification of early esophageal lesions localized and limited only to the mucosa and submucosa may enhance the cure rate for patients with HNSCC [1, 7–11]. Moreover, these esophageal lesions can potentially be completely removed by endoscopic mucosal resection [7, 8, 10, 12]. 

Early SESCC is diagnosed almost exclusively by endoscopic methods [11, 12]. In contrast to standard white light esophagoscopy, which simply observes the macroscopic appearance of mucosal lesions without any enhancement, chromoendoscopy (Lugol's solution chromoendoscopy or methylene blue contact endoscopy) and “electronic chromoendoscopy” (autofluorescence or narrow-band imaging) enable detection of lesions that are not otherwise visible. These methods can be used to accurately assess the extent of the lesions [13–18]. Chromoendoscopy with Lugol's solution can visualize suspicious areas, called the Lugol-voiding lesions (LVLs, also known as the Lugol unstained lesions) and thus detect dysplasias/SCCs that are not normally visible [1, 3, 10, 17, 19, 20].

The aim of this prospective study was to define the benefits of flexible esophagoscopy with chromoendoscopy in the detection of early esophageal carcinomas (second primary carcinomas) in patients with HNSCC. To do so, we used chromoendoscopy with Lugol's solution.

## 2. Material and Methods

### 2.1. Study Design

The study was approved by the Institutional Ethics Committee and performed in accordance with the Declaration of Helsinki, good clinical practice, and applicable regulatory requirements. Informed written consent was obtained from all participants or a legal representative before initiation of any procedure.

A total of 132 patients with newly diagnosed HNSCC underwent chromoendoscopy with Lugol's solution in 2004–2012. Only patients with oral cavity, pharyngeal, and laryngeal carcinomas were included in this prospective study. Patients with iodine allergy were excluded from the study. Patient demographic data were collected, and tumors were staged. The staging included a complete ENT examination, ultrasound and CT scans, and a biopsy of mass lesions. Additional staging procedures were performed when necessary. An individual treatment plan was assigned to each patient after completion of the staging.

### 2.2. Chromoendoscopy

The Lugol chromoendoscopy of the esophagus was performed as an outpatient procedure. Participants were placed in a recumbent position, and a transoral approach was used for the endoscopy. Video endoscopes were used (models GIFQ140, GIFQ145, GIFH180, GIFN180, and GIFV2; Olympus Optical Company, Hamburg, Germany). 

During a conventional examination, the unstained appearance of the esophagus was documented, and the esophagus was evaluated. Then, 15–20 mL of a 3.0% Lugol's iodine solution was sprayed over the entire esophageal mucosa with a spraying catheter (Olympus PW-205V), moving from the lower to the upper esophageal sphincter over the course of 20 seconds. After a 2-minute waiting period, the areas with less-intense staining were photographed; several biopsies were taken from the unstained areas. 

### 2.3. Statistical Analysis

For statistical analysis of age, sex, and treatment success, two-sample *t*-test and Fisher's exact test were used. Results were considered to be statistically significant at *P* < 0.05.

## 3. Results

One hundred and thirty-two subjects with HNSCC were prospectively enrolled in this study (117 men and 15 women). The mean age of the cohort was 57.6 years (range 36–78 years).

**Figure 1:**
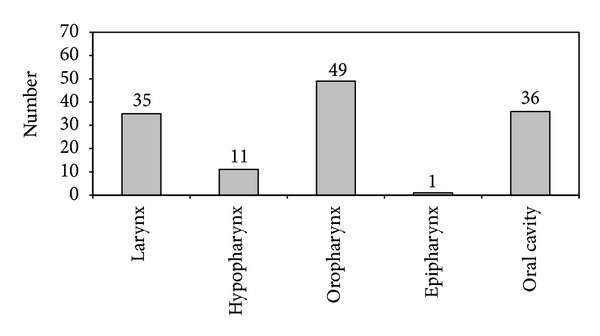
Localization of primary HNSCC in patients screened for the presence of synchronous esophageal pathology by chromoendoscopy.

**Figure 2:**
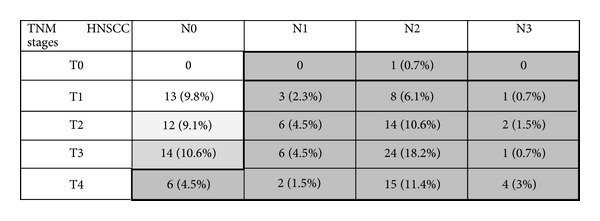
Tumor staging of head and neck cancer according to TNM classification. Stage I: T1N0M0; stage II: T2N0M0; stage III: T3N0M0; stage IV: T4N0-3M0.

**Figure 3:**
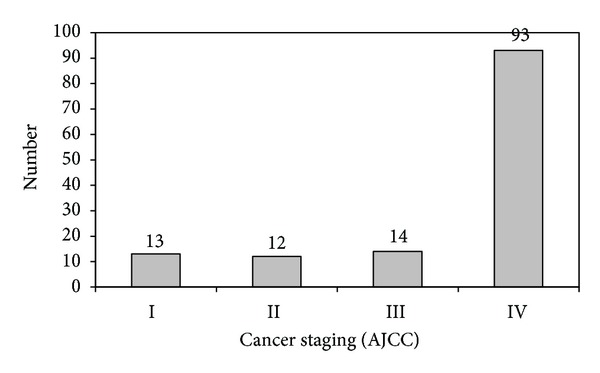
Staging of head and neck carcinomas according to the American Joint Committee on Cancer (AJCC).

The most frequent primary tumor site was oropharyngeal (49/132, 37.1%), followed by the oral cavity (36/132, 27.3%), larynx (35/132, 26.5%), hypopharynx (11/132, 8.3%), and epipharynx (1/132, 0.8%) (Figure 1). Only 9.8% of patients had American Joint Committee on Cancer (AJCC) stage I disease; 9.1% had stage II. The vast majority of the subjects (107/132, 81.1%) and advanced carcinomas had stages III and IV (Figures 2 and 3). Seventy-eight patients reported severe deglutition problems at the time of the HNSCC diagnosis. It was not possible to distinguish whether these problems originated from the head and neck or esophagus. 

Esophagoscopy with chromoendoscopy was performed successfully for all 132 patients. In 25 patients, a percutaneous gastrostomy was performed, due to previously planned surgery for the HNSCC and adjuvant radiotherapy at the same sitting.

**Figure 4:**
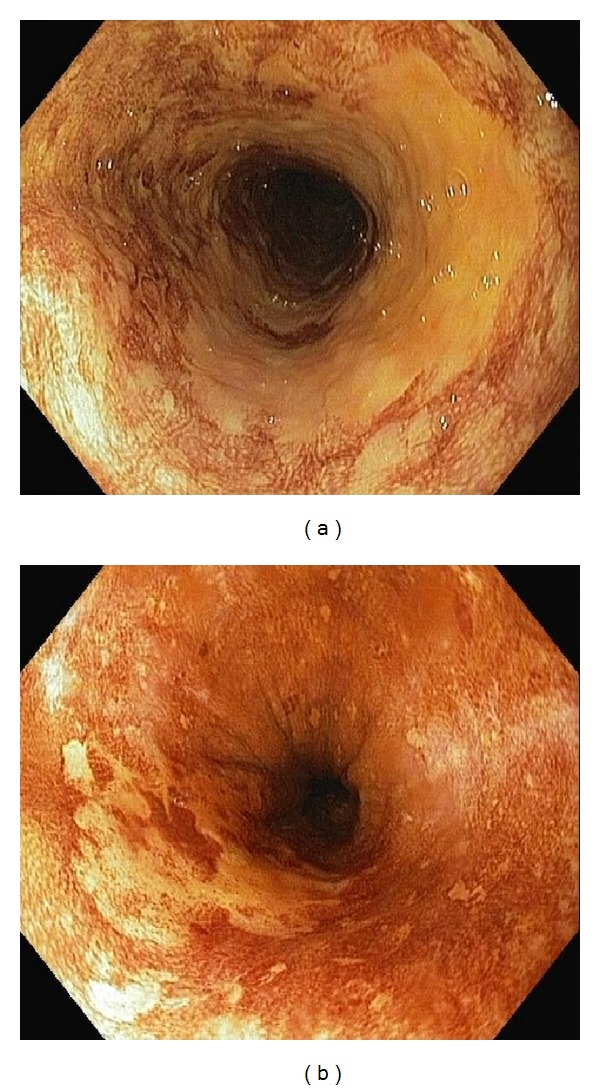
Endoscopic views of the Lugol chromoendoscopy in patients with head and neck cancer. (a) Irregularly shaped multiform LVLs (histologically identified as spinocelullar carcinoma). (b) Single LVL on 7 o'clock position (histologically high-grade dysplasia).

Multiple and no LVLs were discovered in 24 (18.2%) and 108 (81.8%) patients, respectively. A total of 55 biopsy specimens from LVLs were obtained and assessed histopathologically (Figures 4(a), and 4(b)). For these specimens, the diagnoses were as follows: SESCC in two cases, peptic esophagitis in 11 patients, gastric heterotopic mucosa in two patients, hyperplasia in two patients, and dysplasia in three patients (Table 1). Three of the 55 biopsies were described as normal. There was only one case of high-grade dysplasia, which was treated with endoscopic mucosal resection (EMR).

Both the SESCCs were visible during the standard esophagoscopy with white light and were more advanced than T1; the primary HNSCCs were stage IV. Both the SESCCs were also evaluated as LVLs; their boundaries were more visible during the chromoendoscopy. Moreover, the extent of the tumors was found to be greater during chromoendoscopy than had been thought prior to the procedure. In both patients with SESCC, the oncological treatment for HNSCC was changed, and the intended radical surgical treatment was discontinued. 

We experienced complications of chromoendoscopy in one patient. Light bronchospasm that was triggered by aspiration of a small amount of Lugol's solution was treated with standard bronchodilation therapy. The patient was admitted to hospital for observation and discharged the next day. No other complications were observed.

## 4. Discussion

In patients with primary HNSCC, the existence of a second primary tumor in another area of the head or neck, such as the esophagus or the lung, varies from 16% to 36% [1–4, 21–23]. The prevalence of synchronous esophageal cancer in patients with head and neck cancer varies from 5.1% to 12.5% [5]. Synchronous multiple primary cancers in the esophagus and the head and neck region adversely affect survival and quality of life, because the mortality rate for patients with a second cancer, especially cancer in the lung and esophagus, is as high as 90% at 2 years after detection [1, 5].

The highest incidence of SESCC was found in the Chinese province of Linxian, where the incidence of SESCC is 700 cases per 100,000 people (this is approximately 350 times higher than the incidence of esophageal SCC in the Czech Republic) [6, 11, 24]. Dubuc et al., in a large European study, discovered esophageal neoplastic lesions in 9.8% of patients with HNSCC [25], whereas the overall incidence of SESCC in the Czech Republic is quite low, approximately two per 100,000 people [24].

The etiology of HNSCC and SESCC is associated predominantly with smoking and drinking [3, 6, 10, 26–28]. This may be explained by the concept of “field cancerization” proposed by Slaughter et al., in which repeated exposure to carcinogens leads to an accumulation of genetic alterations that results, ultimately, in the development of multiple and independent cancers [1, 2]. It is the reason why the upper aerodigestive tract should be examined as part of the staging workup in patients with HNSCC even in patients without symptoms of deglutition disorders [1, 10, 14, 15, 20, 22] and why particular attention should be focused on high-risk groups, such as patients with primary HNSCC.

Early detection of SESCC with using neoplasia classification based on cytological and architectural severity and invasion status are essential for effective treatment, and the prognosis strongly depends on tumor stage at the time of diagnosis [1–3, 6, 9–13, 26, 27, 29–32]. One of the most important reasons for why early SESCCs are not detected is the fact that there is a tendency to carry out cursory examinations of the esophagus using white light only [7]. “Biologic endoscopy” or “detailed endoscopy” can be used for early detection of tumors in the upper aerodigestive tract, because these techniques enable the visualization of lesions that are not otherwise apparent and can provide greater insight into the behavior of target lesions [1, 4, 13–17, 29–31].

Iodine staining of squamous cell epithelium is one of the oldest staining techniques; it was first described by Schiller in 1933 for the detection of early carcinoma of the cervix [32]. Lugol's staining patterns correlate well with the degree of glycogen within squamous epithelium, and dysplastic epithelium can be visualized as LVLs [1, 5, 8, 17, 33]. Chromoendoscopy is inexpensive and can be performed easily by a gastroenterologist; no special tools or light sources are necessary [1].

Muto et al. reported that 55% of HNSCC patients with many irregularly shaped multiform LVLs had synchronous SESCC. Moreover, Fukuhara et al. reported that 85.7% of HNSCC patients with metachronous SESCC had many irregularly shaped multiform LVLs [1, 3]. In contrast to these findings, we observed LVLs in only 24 patients (18.2%), and we found no early SESCCs and only three dysplasias (one of which was treated with endoscopic mucosal resection).

The aim of our study was to define the incidence of esophageal lesions by chromoendoscopy with Lugol's solution in patients with newly diagnosed HNSCC. Our idea was to detect synchronous early esophageal carcinomas in this high-risk group of patients, in the group in which no or minimal deglutition symptoms are present. From this perspective, our results did not meet our expectations, since we did not find any early SESCCs. However, we believe that detailed endoscopy techniques can improve detection of superficial mucosal areas with dysplasia or early carcinoma and should therefore be considered standard methods during flexible esophagoscopy. This is especially true for high-risk patient groups, such as those with primary HNSCC. Advanced endoscopic methods should be used not only for staging of the primary HNSCC tumor, but also for followup to detect possible second metachronous primary esophageal tumors.

## 5. Conclusions

Patients with HNSCC represent a high-risk group for the development of SESCC. Thus, esophagogastrofibroscopy should be performed to detect possible synchronous esophageal carcinomas in these patients.

Although only two patients with synchronous primary carcinomas were found among the patients with newly diagnosed HNSCC in this study, esophagoscopy and better some of advanced endoscopic methods should be recommended after detection of HNSCC to exclude secondary esophageal carcinoma or dysplasia. Staining of the esophagus with Lugol's solution is an easy and inexpensive option and can be done in most of gastroenterology offices. 

## Figures and Tables

**Table 1:** Histopathological diagnosis of mucosal biopsies.

Pathology	*N*
Squamous cell carcinoma	2
High-grade dysplasia	1
Low-grade dysplasia	2
Peptic esophagitis	10
Gastric heterotopic mucosa	2
Hyperplasia	2
Hyperkeratosis	1
Acanthosis	1
Normal mucosa	3

Total	24
